# Linoleic Acid Attenuates Denervation-Induced Skeletal Muscle Atrophy in Mice through Regulation of Reactive Oxygen Species-Dependent Signaling

**DOI:** 10.3390/ijms23094778

**Published:** 2022-04-26

**Authors:** Myung-Hun Lee, Jin-Ho Lee, Wan-Joong Kim, Seo Ho Kim, Sun-Young Kim, Han Sung Kim, Tack-Joong Kim

**Affiliations:** 1 Division of Biological Science and Technology, Yonsei University, Wonju 26493, Korea; onlyonce05@naver.com (M.-H.L.); drlogos@naver.com (J.-H.L.); logix328@naver.com (W.-J.K.); seereal1@naver.com (S.H.K.); still378@naver.com (S.-Y.K.); 2 Department of Biomedical Engineering, Yonsei University, Wonju 26493, Korea; hanskim@yonsei.ac.kr; 3 Research & Development Center, Doctor TJ Co., Ltd., Wonju 26493, Korea

**Keywords:** antioxidant, linoleic acid, muscle atrophy, oxidative stress, sciatic denervation

## Abstract

Muscle atrophy is a major muscle disease, the symptoms of which include decreased muscle volume leading to insufficient muscular support during exercise. One cause of muscle atrophy is the induction of oxidative stress by reactive oxygen species (ROS). This study aimed to identify the antioxidant mechanism of linoleic acid (LA) in muscle atrophy caused by oxidative stress. H_2_O_2_ has been used to induce oxidative stress in myoblasts in vitro. C2C12 myoblasts treated with H_2_O_2_ exhibited decreased viability and increased ROS synthesis. However, with LA treatment, the cells tended to recover from oxidative effects similar to those of the control groups. At the molecular level, the expression of superoxide dismutase 1 (SOD1), Bax, heat shock protein 70 (HSP70), and phosphorylated forkhead box protein O1 was increased by oxidative stress, causing apoptosis. LA treatment suppressed these changes. In addition, the expression of *MuRF1* and *Atrogin-1/MAFbx* mRNA increased under oxidative stress but not in the LA-treated group. Sciatic denervation of C57BL/6 mice manifested as atrophy of the skeletal muscle in micro-computed tomography (micro-CT). The protein expression levels of SOD1, HSP70, and MuRF1 did not differ between the atrophied muscle tissues and C2C12 myoblasts under oxidative stress. With LA treatment, muscle atrophy recovered and protein expression was restored to levels similar to those in the control. Therefore, this study suggests that LA may be a candidate substance for preventing muscle atrophy.

## 1. Introduction

Muscle atrophy is a disease in which muscle tissue decreases as a result of damage to myoblasts [1,2]. The common causes of atrophy are muscle loss from physical damage, long periods of being bedridden, low-gravity conditions such as being in space, and severe diseases such as Lou Gehrig’s disease [3,4]. Regular contraction activity is important for maintaining the structural and physiological functions of skeletal muscles. Muscle loss is promoted by continuous skeletal muscle inactivity. This leads to reduced protein synthesis and increased ubiquitin-protein degradation [5,6].

Exposure of skeletal muscle to hydrogen peroxide, a type of reactive oxygen species (ROS), has been reported to stimulate protein catabolism in skeletal muscle by upregulating the ubiquitin-binding system, in addition to interfering with cell signaling pathways that promote protein synthesis [7,8]. Redox signals are important regulators of cell signaling pathways that control protein synthesis and proteolysis in skeletal muscles. According to reports, oxygen stress via ROS induction stimulates a proteolysis process in the nucleus and the activity of calpine and caspase 3. Ultimately, muscle fibers degrade and protein deformation and decomposition are accelerated [9].

Superoxide dismutase 1 (SOD1) is an antioxidant enzyme that can neutralize anion superoxide radicals in the cell [10,11]. In addition, heat shock proteins (HSPs) are essential factors in protein homeostasis and possess chaperone properties. They assist in the folding and intracellular transport of proteins, facilitating the recovery or utilization of partially denatured proteins upon exposure to various stress factors. The heat shock protein 70 (HSP70) protects cells from multiple stresses, and its expression is vital for cell survival under stressful conditions. Cells lacking HSP70 are unable to endure the effects of stress stimuli and undergo apoptosis [12,13]. Forkhead box protein O (Foxo), a transcription factor, regulates cell proliferation, the cell cycle, and cell survival. In particular, in skeletal muscles, Foxo proteins contribute to determining muscle size through transcriptional regulation of atrogen products such as Atrogen-1/MAFbx and muscle ring finger protein 1 (MuRF-1), which act on E3 ubiquitin-binding enzymes [14,15,16]. Because MuRF1 and Atrogin-1/MAFbx proteins have been shown to induce muscle cell death, the regulation of FoxO1 by external stimuli can play a critical role in muscle atrophy. MuRF1 is a ring finger domain protein expressed in myoblasts [17]. Increased MuRF1 and Atrogin-1/MAFbx expression lead to the ubiquitination of vital proteins, affecting myoblast survival and inducing cell death [18].

Linoleic acid (LA) has been reported to prevent arteriosclerosis and cardiovascular hypertension and maintain homeostasis [19,20,21]. In addition, CIS9-TRANS11-CLA, a major conjugated linoleic acid (CLA) isomer, is known to promote health as a natural derivative of LA with nutritional benefits, including antidiabetic and anticancer properties, immune system modulatory effects, and weight-protectors. Another important CLA isomer, CIS10-TRANS12-CLA, has also been reported to exhibit anti-obesity effects [22]. The effects of LA and CLA in animal models of muscle atrophy induced by high-fat diet obesity or increased bone density in infants have been reported [23,24]. However, the biological effects and mechanisms of LA on muscle atrophy caused by oxidative stress are yet to be elucidated.

In this study, we examined the effect and mechanism of action of LA in H_2_O_2_-induced C2C12 myoblasts and the sciatic denervation model of C57BL/6 mice. 

## 2. Results

### 2.1. Linoleic Acid Attenuates the Cell Viability Decrease and Oxidative Stress-Mediated Apoptosis in C2C12 Myoblasts

**Figure 1:**
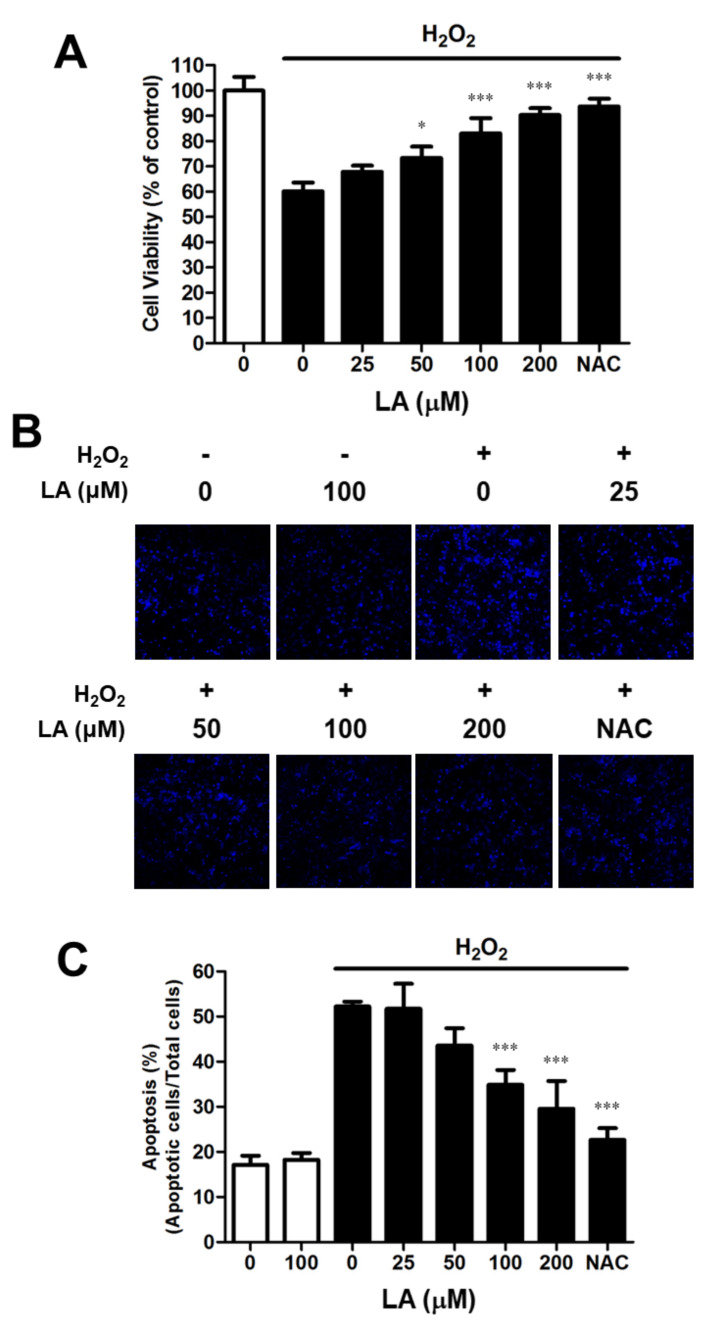
Linoleic acid prevents oxidative stress-induced cell death in C2C12 myoblasts. C2C12 myoblasts were seeded, and the medium was then replaced with serum-free DMEM containing LA (0–200 mM) for 24 h. After pre-incubation, C2C12 myoblasts were treated with 1 mM H_2_O_2_ for 2 h. The cell viability was assessed by using the EZ-Cytox cell viability kit. Absorbance at 450 nm was measured using a microplate reader. (**A**) Effect of LA on H_2_O_2_-induced cell viability in C2C12 myoblasts. The cell viability was calculated according to the following equation: cell viability (%) = [(absorbance of the H_2_O_2_-treated sample/absorbance of the H_2_O_2_-untreated control) × 100]. Each value indicates the mean ± SD (*n* = 5). (**B**) Effect of LA on apoptosis by oxidative stress in C2C12 myoblasts. C2C12 myoblasts were fixed with 4% *para*-formaldehyde in PBS for 30 min and mounted using a mounting medium with DAPI. Imaging data were acquired on a confocal microscope. DAPI staining was detected at wavelengths of 358 and 461 nm. (**C**) Quantitating apoptotic cells. Apoptotic cells were measured using the Image J software. Apoptosis was calculated according to the following equation: apoptosis (%) = [(number of apoptotic cells/number of total cells) × 100]. Each value indicates the mean ± SD (*n* = 4). * *p* < 0.05 and *** *p* < 0.001. vs. H_2_O_2_ alone. The effect of LA was compared with that of 2 mM *N*-acetyl-l-cysteine (NAC).

C2C12 myoblasts were treated with LA (0–200 µM) for 24 h to ascertain the effect of LA on oxidative stress. Additionally, they were incubated for 2 h with 2 mM H_2_O_2_ to induce oxidative stress. The cell viability of the 25, 50, 100, and 200 µM LA-treated group increased compared with the group treated with H_2_O_2_ only (67.78 ± 2.46, 73.30 ± 4.49, 83.03 ± 6.02, 90.23 ± 2.80 vs. 60.11 ± 3.49%, respectively) (Figure 1A). Due to concerns about cell viability in an environment with oxidative stress, LA concentrations in the range of 0–200 µM were tested to measure cytotoxicity in C2C12 myoblasts. We did not find LA-induced cytotoxicity at any of the concentrations tested (data not shown). DAPI staining was performed to assess apoptosis in response to oxidative stress. Apoptosis induced by H_2_O_2_ increased to 35.09% compared to that in the untreated control (11.45%). Pretreatment for 23 h with LA (25, 50, 100, and 200 µM) reduced apoptosis (Figure 1B,C). This experiment confirmed that cell death caused by apoptosis affected cell viability; treatment with LA prevented oxidative-stress-induced apoptosis in C2C12 myoblasts.

### 2.2. Linoleic Acid Decreases Oxidative Stress-Induced MuRF1 and Atrogin-1 mRNA Expression in C2C12 Myoblasts

**Figure 2:**
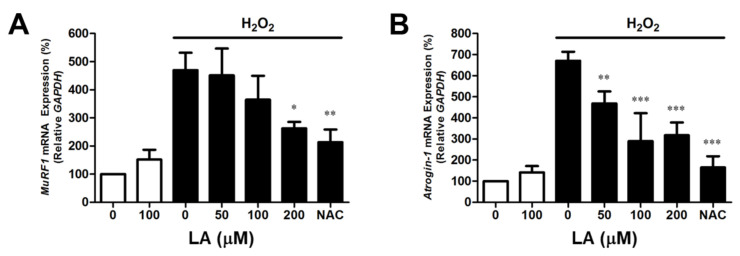
Linoleic acid decreases *MuRF1* and *Atrogin-1/MAFbx* mRNA expression in oxidative stress-induced C2C12 myoblasts. C2C12 myoblasts were cultured with serum-free DMEM containing LA (0–200 µM). Cell lysis and mRNA extraction were performed using Tri-reagent. cDNA was synthesized according to the manufacturer’s instructions. mRNA expression was analyzed by using SYBR Green 1 and a LightCycler ® 96 instrument. (**A**) *MuRF1* mRNA expression. Each value indicates the mean ± SD (*n* = 6). (**B**) *Atrogin-1/MAFbx* mRNA expression. Each value indicates the mean ± SD (*n* = 6). * p < 0.05, ** p < 0.01, *** p < 0.001 vs. H_2_O_2_ alone. The effect of LA was compared with that of 2 mM NAC.

Real-time PCR was used to analyze mRNA expression to estimate *MuRF1* and *Atrogin-1/MAFbx* gene transcription. H_2_O_2_-treated C2C12 myoblasts showed increased *MuRF1* mRNA expression levels (469.59 ± 61.96%). However, pre-treatment with LA (50, 100, and 200 µM) reduced *MuRF1* mRNA expression to 451.42 ± 95.36, 364.92 ± 84.89, and 262.59 ± 23.14% (Figure 2A). In addition, the *Atrogin-1/MAFbx* mRNA expression levels also increased (671.07 ± 42.73%). Pre-treatment with LA (50, 100, and 200 µM) reduced *Atrogin-1/MAFbx* mRNA expression to 468.35 ± 57.45, 289.79 ± 132.81, and 317.80 ± 60.38%, respectively (Figure 2B). This treatment suggests that LA suppresses the expression of mRNAs related to cell death and muscle atrophy genes, such as *MuRF1* and *Atrogin-1/MAFbx,* caused by oxidative stress in C2C12 myoblasts.

### 2.3. Linoleic Acid Decreases Intracellular Reactive Oxygen Species Synthesis in C2C12 Myoblasts

**Figure 3:**
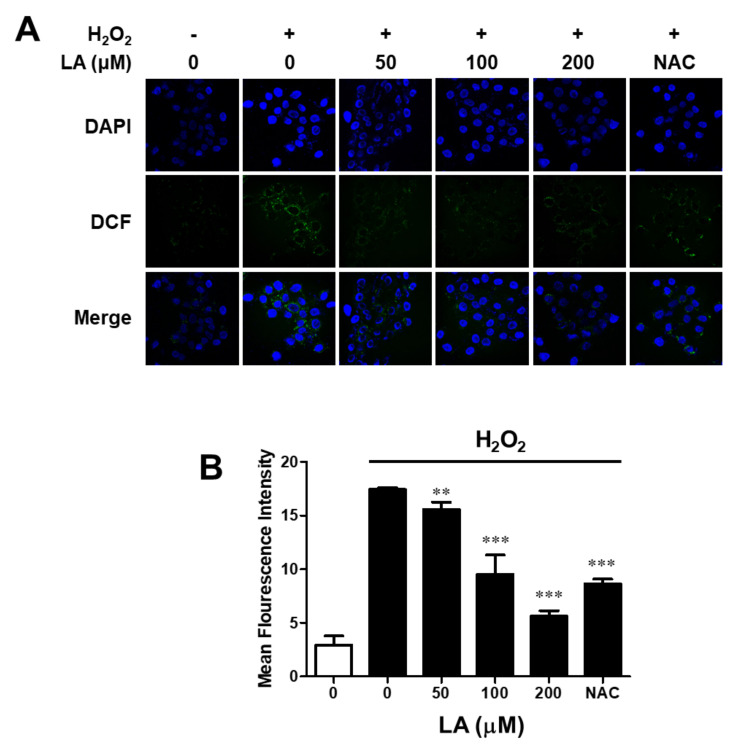
Linoleic acid decreases intracellular ROS synthesis in oxidative stress-induced C2C12 myoblasts. For the estimation of intracellular ROS, DCF was detected using confocal microscopy. (**A**) The effect of LA on intracellular ROS in C2C12 myoblasts identified using a confocal microscope. C2C12 myoblasts were pre-incubated in serum-free DMEM with LA (0–200 µM) for 24 h, and the medium was replaced with 1 mM H_2_O_2_ for 2 h. After incubation, C2C12 myoblasts were treated with 10 µM DCF-DA in PBS for 30 min, fixed with 4% *para*-formaldehyde in PBS for 30 min, and mounted using a mounting medium with DAPI. DCF was detected at wavelengths of 485 and 535 nm. DAPI was detected at wavelengths of 358 and 461 nm. The effect of LA was compared with that of 2 mM NAC. (**B**) Quantitating intracellular ROS synthesis. A graph showing average intensity of fluorescence in various GFPs, observed with a confocal laser scanning microscope in the C2C12 myoblast. Data are represented as mean ± SD (*n* = 3). ** *p* < 0.01, *** *p* < 0.001 vs. H_2_O_2_ alone.

Muscle cell injury caused by ROS is associated with various muscular disorders and pathogenic conditions. The generation of intracellular ROS in myoblasts was measured with 2′,7′–dichlorofluorescein diacetate (DCF-DA). As shown in Figure 3, 2′,7′–dichlorofluorescein (DCF) was found in the cytoplasm of H_2_O_2_-treated cells. The increase in fluorescence was proportional to the level of the intracellular ROS synthesized. However, the fluorescence intensity of the LA-treated group was similar to that of the control group. LA demonstrated antioxidant capacity against ROS synthesis in C2C12 myoblasts.

### 2.4. Effect of Linoleic Acid on the Altered Intracellular Protein Levels Induced by Oxidative Sress in C2C12 Moblasts

**Figure 4:**
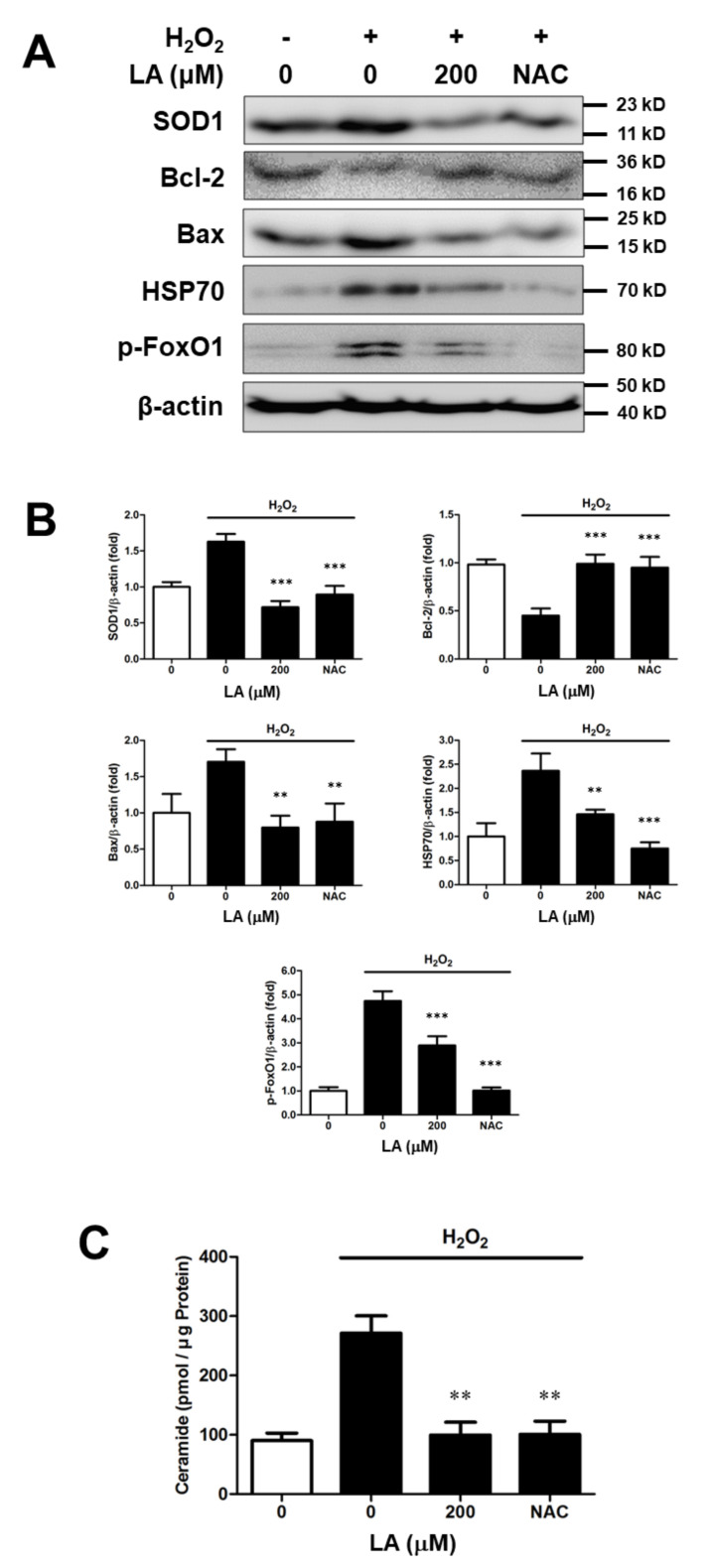
Effect of linoleic acid on intracellular protein and ceramide level of oxidative stress-induced C2C12 myoblasts. C2C12 myoblasts were seeded and cultured in serum-free DMEM with or without 200 mM LA. Protein expression level as analyzed by SDS-PAGE. Cell lysis and protein extraction were conducted using the PRO-PREP protein extraction kit. (**A**) Effect of LA on SOD1, Bcl-2, Bax, HSP70 and p-FoxO1 protein expression level by oxidative stress in C2C12 myoblasts. (**B**) Relative density SOD1, Bcl-2, Bax, HSP70 and p-FoxO1 protein expression level. Each value indicates the mean ± SD (*n* = 3). (**C**) Effect of LA on ceramide levels under H_2_O_2_-induced oxidative stress in C2C12 myoblasts. Ceramide levels as analyzed by HPTLC. Cells were lysed in 0.2 N NaOH. Lipids were extracted using a methanol/chloroform/1 M NaCl solution. Lipids were separated using a silica gel TLC plate and analyzed by HPLC. Each value indicates the mean ± SD (*n* = 3). ** *p* < 0.01, *** *p* < 0.001 vs. H_2_O_2_ alone. The effect of LA was compared with that of 2 mM NAC.

To evaluate protein expression altered by oxidative stress at the molecular level, immunoblotting and quantitative analysis of proteins were conducted. The 1 mM H_2_O_2_-treated group exhibited increased levels of SOD1 expression in C2C12 myoblasts. The expression of Bax, HSP70, and p-FoxO1 increased under oxidative stress. However, the 200 µM LA-treated group showed lower protein expression than the group treated with H_2_O_2_ only. In contrast, the Bcl-2 expression levels decreased upon oxidative stress, but recovered to baseline levels after 200 µM LA treatment (Figure 4A,B). These results imply that oxidative stress attacks C2C12 myoblasts to induce cell death, but LA protects C2C12 myoblasts from oxidative-stress induced cell death.

### 2.5. Linoleic Acid Decreases Intracellular Ceramide Level in Oxidative Stress-Induced C2C12 Myoblasts

We performed quantitative analysis of endogenous ceramide on oxidative stress by H_2_O_2_ in C2C12 myoblasts. The HPTLC analysis method was used to separate ceramide from the whole lipid extract. HPLC was used to quantify ceramides in C2C12 myoblasts. Ceramide increased by H_2_O_2_ in C2C12 myoblasts. In contrast, ceramide levels decreased in the presence of LA (200 µM) (Figure 4C). Therefore, LA treatment decreased the ceramide content, thereby suppressing apoptosis.

### 2.6. Linoleic Acid Reduces Muscle Volume on Sciatic Denervation-Induced Muscle Atrophy of C57BL/6 Mice

**Figure 5:**
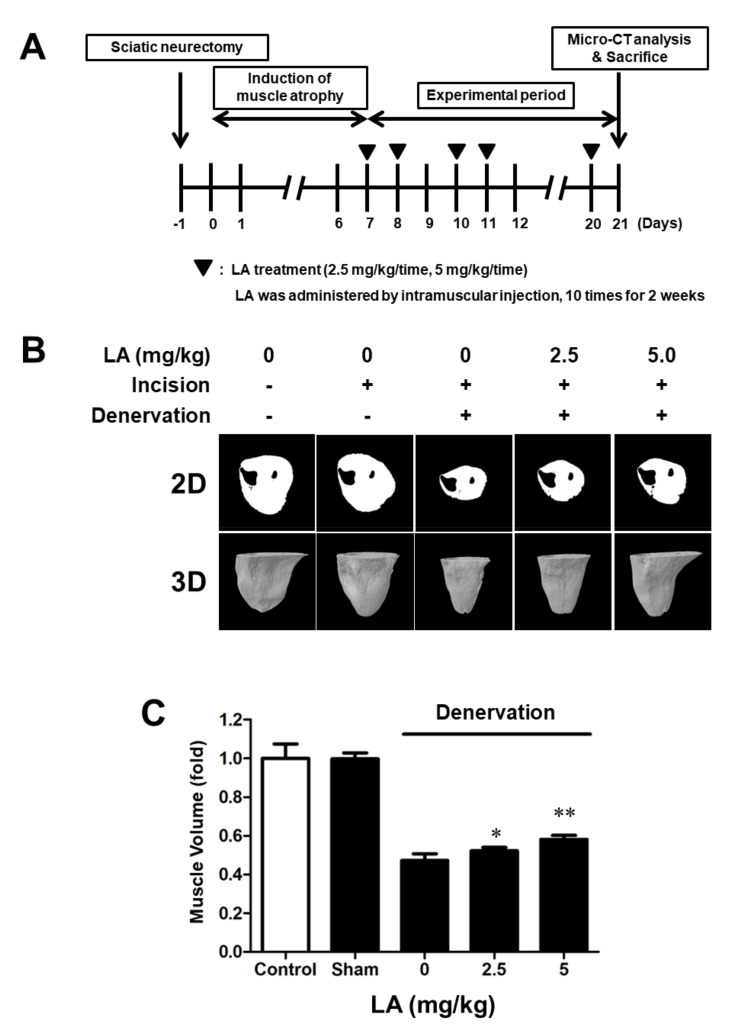
Linoleic acid treatment prevents denervation-induced muscle atrophy in C57BL/6 mice. (**A**) Muscle atrophy was induced in C57BL/6 mice by sciatic denervation. Evaluation of muscle volume and analysis of two-dimensional (2D) and three-dimensional (3D) models were conducted 21 days after sciatic denervation. LA (0–5 mg/kg) was administered by intramuscular injection. (**B**) Effects of LA on disuse atrophy by sciatic denervation in mice. (**C**) Evaluation of muscle volume after sciatic denervation. Evaluation of muscle volume after treatment with LA (0–5 mg/kg). Each value indicates the mean ± SD (*n* = 6); * *p* < 0.05, ** *p* < 0.01 vs. sciatic denervation alone.

The sciatic nerve was surgically removed to induce muscle atrophy in C57BL/6 mice [25,26,27]. The mice could not use the sciatic-denervated leg and had reduced mobility. After 21 days, the muscle volume of C57BL/6 mice was evaluated using micro-CT and 2D and 3D models (Figure 5A). The muscle size of sciatic-denervated mice was reduced compared to that of the mice in the control group. In contrast, LA (2.5- and 5 mg/kg)-treated mice showed a recovery in muscle volume (Figure 5B,C). This result implies that considerable disuse of the muscle by sciatic denervation induced muscle atrophy, and LA prevented and recovered the atrophy in C57BL/6 mice. 

### 2.7. Linoleic Acid Decreases SOD1, HSP70, and MuRF1 Protein Expression on Sciatic Denervation-Induced Muscle Atrophy of C57BL/6 Mice

**Figure 6:**
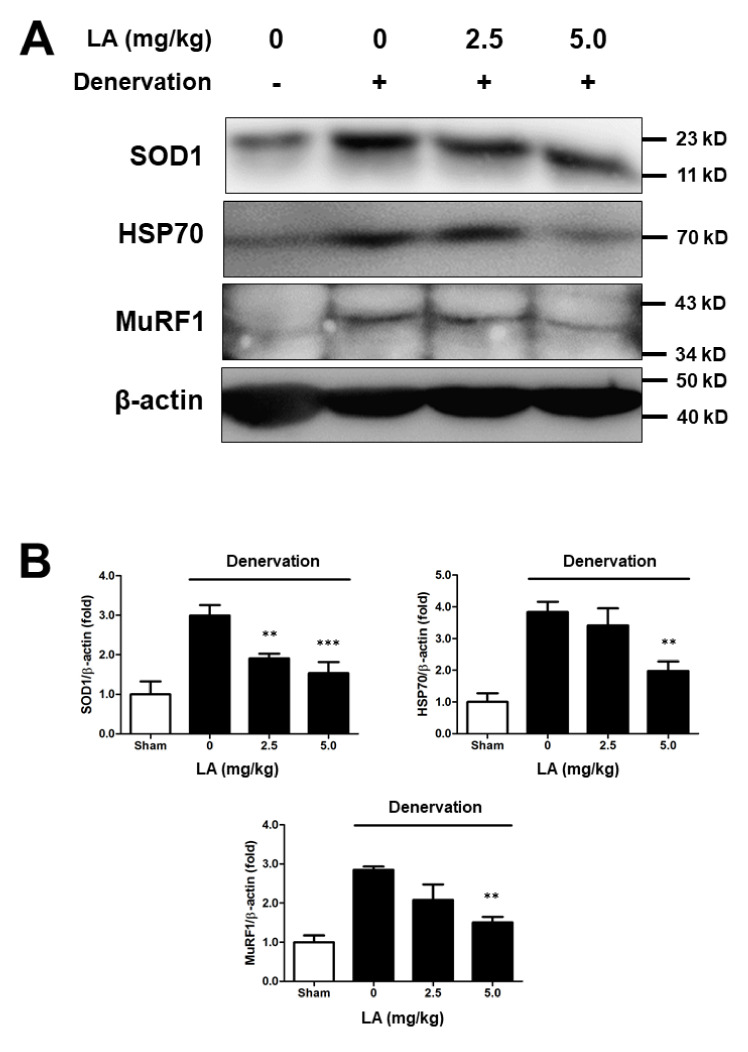
Linoleic acid decreases SOD1, HSP70, and MuRF1 protein expression in sciatic denervation-induced muscle atrophy of C57BL/6 mice. Muscle atrophy was induced in C57BL/6 mice by sciatic denervation. Protein expression level as analyzed by SDS-PAGE. Lysis of muscle tissue and protein extraction was performed using the PRO-PREP protein extraction kit. (**A**) Effect of LA on SOD1, HSP70, and MuRF1 protein expression level by sciatic denervation-induced muscle atrophy in mice. (**B**) Relative density of SOD1, HSP70, and MuRF1 protein expression level. Each value indicates the mean ± SD (*n* = 6); ** *p* < 0.01, *** *p* < 0.001 vs. sciatic denervation alone.

To understand the causes of muscle atrophy, we performed immunoblotting of tissues from mice under oxidative stress. Sciatic-denervated mice had increased SOD1, HSP70, and MuRF1 protein levels, and LA (2.5 and 5 mg/kg) treatment recovered the protein levels to baseline (Figure 6A,B). These results were similar to those observed in C2C12 myoblasts under oxidative stress. Therefore, muscle atrophy caused by sciatic denervation was induced by oxidative stress in mice, and LA prevented and reversed this muscle atrophy.

## 3. Discussion

This study identifies a new pharmaceutical candidate to prevent and treat muscle atrophy, a debilitating muscle disease [28]. Given that the action of muscles involves contraction and relaxation, muscular strength decreases owing to the loss of myoblasts [29]. Every physical activity of animals, including humans, depends on muscular ability; muscular dysfunction, affects exercise, daily life, and even social life, negatively affecting the quality of life. Moreover, because the quality of modern human life is a function of outdoor activities and social welfare, muscular health is especially essential. Muscle atrophy can be caused by direct damage to the muscle, long periods of immobilization, and various muscle-related diseases. Therefore, muscle atrophy can easily affect any individual. In this study, sciatic-denervated mice showed decreased muscle volume due to muscle disuse. Therefore, we aimed to determine the effects of LA on atrophied muscle. LA significantly recovered the muscle volume in sciatic denervated mice (Figure 5). 

Various factors are known to be involved in muscle atrophy. ROS are also associated with multiple muscular disorders [8,30,31]. Petry et al. proposed the oral supplementation of l-glutamine plus l-alanine and L-alanyl-l-glutamine as therapeutic and effective nutritional alternatives to attenuate the deleterious effects of skeletal muscle protein degradation induced by muscle disuse [32]. The main cause for myoblasts damage in the body is oxidative stress, such as that mediated by ROS [33,34]. Muscle is the tissue most relevant to oxidative functions; therefore, it is logical to assume that it is subjected to the highest oxidative stress among the various tissues of the human body. In cases of extreme exercise, an increase in ROS production leads to muscle injury [34]. Oxygen influx occurs rapidly when muscles contract. An individual becomes more susceptible to muscle damage [35,36,37]. The sciatic denervation state is a destroyed arrangement of myosin filaments caused by an increase in ROS and degradation of muscle-specific proteins [35].

Confocal microscopy was used to determine the amount of intracellular ROS in LA-treated C2C12 myoblasts, and the amount of DCF significantly decreased in LA-treated C2C12 myoblasts (Figure 3). Based on these results, anti-oxidative protection may be a useful strategy to prevent oxidative injury or delay the progression of related diseases, such as muscular disorders.

The pro-apoptotic cell signaling pathway of myoblasts induced by oxidative stress is an important factor underlying muscle atrophy [38]. Therefore, we assessed the effects of LA by inducing cell death in myoblasts and confirmed that LA protected C2C12 myoblasts from oxidative stress and recovered cell viability in the presence of H_2_O_2_ (Figure 1A). Moreover, C2C12 myoblasts exposed to oxidative stress showed a decreased tendency for apoptosis with LA treatment alone (Figure 1B,C). Therefore, our results suggest that apoptosis of myoblasts via oxidative stress is a key cause of muscle atrophy and that LA can protect myoblasts from oxidative stress-induced cell death.

We investigated the mechanism underlying the relationship between muscle atrophy and ROS levels. SOD1 acts as a catalyst for catalase through a chemical reaction activated by naturally occurring superoxide radicals such as molecular oxygen and hydrogen peroxide. Therefore, SOD1 has the ability to eliminate ROS in cells [35,39]. We observed changes in SOD1 expression after LA-treatment under oxidative stress using immunoblot analysis. As a result, SOD1 expression was increased in H_2_O_2_-treated C2C12 myoblasts, and SOD1 expression decreased with LA treatment (Figure 4). LA-injected C57BL/6 mice after sciatic denervation showed the same characteristics in vitro (Figure 6). Bcl-2, an anti-apoptotic factor, exhibited reduced protein expression in an oxidative stress environment, which was recovered with LA treatment. However, Bax, an apoptotic factor, showed an opposite pattern (Figure 4). HSP70 prevents cell damage caused by stress stimuli, including oxidative stress [35]. HSP expression increases in response to stressors. In our study, HSP70 expression increased in H_2_O_2_-treated C2C12 myoblasts and decreased upon treatment with LA (Figure 4). Mice with induced muscle atrophy exhibited increased HSP70 expression in vivo, but mice injected with LA recovered HSP70 expression to levels similar to those in the control group (Figure 6). Therefore, significant changes in HSP70 levels due to muscle atrophy indicate that HSP70 activity may protect muscles through several cellular responses [40]. The phosphorylation of FoxO1 has been identified in myoblasts under induced oxidative stress. The phosphorylation of FoxO1 was increased by H_2_O_2_ exposure and decreased by LA treatment (Figure 4). Recent studies have reported that increased expression of p-FoxO1 leads to increased expression of MuRF1 and Atrogin-1 in myoblasts [41,42,43]. 

MuRF1 is a ring finger protein containing a zinc finger domain, which leads to cell death by ubiquitination of proteins within myoblasts [44,45]. In addition, transcriptional regulation of *Atrogin-1/MAFbx* mRNA is induced during muscle atrophy, similar to *MuRF1* [46]. The increase in *MuRF1* or *Atrogin-1/MAFbx* expression by external stimuli can be thought of as one cause of muscle atrophy through the reduction in muscle volume due to the death of myoblasts. In this study, *MuRF1* and *Atrogin-1/MAFbx* mRNA levels increased under oxidative stress in C2C12 myoblasts, whereas treatment with LA (0–200 µM) decreased *MuRF1* and *Atrogin-1/MAFbx* mRNA levels in a dose-dependent manner (Figure 2). In addition, C57BL/6 mice with muscle atrophy induced by sciatic denervation expressed and regulated the MuRF1 protein in the same manner (Figure 6). Although protein expression analysis of Atrogin-1/MAFbx in sciatic-denervated animals has not been performed, our results based on the mRNA expression results suggest that LA inhibits muscle atrophy through the regulation of MuRF1 or Atrogin-1/MAFbx.

Ceramides are sphingolipid mediators that are involved in stress-inducible apoptosis. Various stress factors leading to apoptosis have been reported to increase ceramide levels in several cell types, including myoblasts [47,48]. During myoblast differentiation, ceramides induce the expression of myogenic transcription factors. In addition, ceramide inhibits myogenin synthesis and myotube formation [49], and several studies have identified the relationship between ceramide and various muscular diseases [50,51]. Induction of oxidative stress in C2C12 myoblasts increased ceramide levels, whereas ceramide decreased in the presence of LA (Figure 4). Therefore, this result suggests that LA protects against cell damage by inhibiting ceramide synthesis in ROS-induced C2C12 myoblasts.

Antioxidants are widely used to protect cells from ROS-induced damage. *N*-acetyl-l-cysteine (NAC) is sold as a dietary supplement that commonly contains antioxidant benefits [52]. Recent studies have reported the protective effect of NAC on muscle damage in mouse models characterized by high oxidative stress, functioning as a potent antioxidant [53,54]. Recently, Michelucci et al. reported that two months of NAC treatment starting at two months of age, when mitochondrial and fiber damage was minimal, reduced the formation of unstructured and contracture cores, improved muscle function, and decreased mitochondrial damage [54]. In another study, rats treated with the lipid-soluble antioxidant vitamin E showed approximately 20% improvement of muscle atrophy [55,56].

Our previous study showed that *Oenothera odorata* (*O. odorata*) root extract is an effective countermeasure to reduce the muscle mass loss induced by microgravity and sciatic denervation [35,57]. Many studies have reported that *O. odorata* comprises 65–75% linoleic acid, 7–10% γ-linolenic acid (GLA); oleic, palmitic, and stearic acids; and the steroids campesterol and β-sitosterol [58,59]. Therefore, results in this study indicate that linoleic acid, the main component of *O. odorata*, improves skeletal muscle atrophy. 

**Figure 7:**
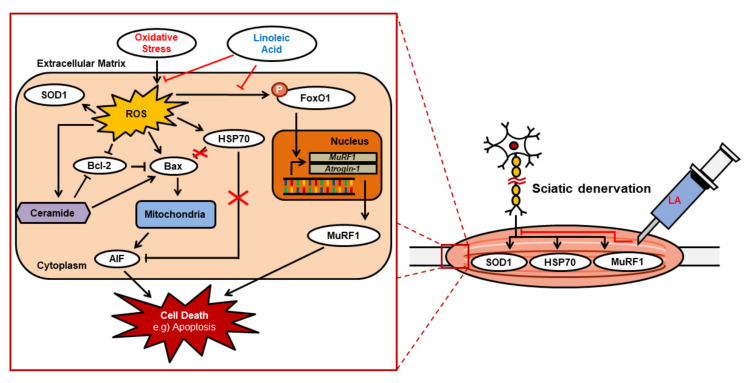
Mechanism of action for linoleic acid on muscle atrophy induced by oxidative stress in vitro and in vivo.

In summary, muscle atrophy was induced in C57BL/6 mice by sciatic denervation to determine whether LA inhibits muscle atrophy. Micro-CT, 2D, and 3D models demonstrated that LA increased muscle volume after sciatic denervation. In addition, we confirmed that ROS-related cellular signaling, including SOD1, Bax, Bcl2, p-FoxO-1, HSP70, ceramide, MuRF1, and Atrogin-1/MAFbx are affected by LA under oxidative stress (Figure 7). Our results showed that antioxidants could be therapeutic agents for muscle atrophy. 

## 4. Materials and Methods

### 4.1. Materials

Penicillin-streptomycin was obtained from Lonza (Walkersville, MD, USA). The EZ-Cytox Cell Viability Kit was purchased from Daeil Lab (Seoul, Korea). LA, *N*-acetyl-l-cysteine (NAC), 2′,7′-dichlorofluorescein diacetate (DCF-DA), *ortho*-phthaldialdehyde (OPA), chloroform, and trypsin-EDTA solutions were purchased from Sigma-Aldrich (St. Louis, MO, USA). Phosphate-buffered saline (PBS) was purchased from Gibco Life Technologies Inc. (Rockville, MD, USA). Antibody against HSP70 was purchased from Enzo Life Sciences (Aargau, Switzerland). Antibodies against Bcl-2, Bax, phospho-FoxO1, β-actin, anti-rabbit IgG, and anti-mouse IgG were purchased from Cell Signaling Technology (Danvers, MA, USA). Antibodies against SOD1 and MuRF1 were purchased from Santa Cruz Biotechnology Inc. (Dallas, TX, USA). Vectashield mounting medium containing DAPI was purchased from Vector Laboratories (Burlingame, CA, USA). HPLC-grade methanol and HPTLC silica gel plates were purchased from Merck (Darmstadt, Germany). Ceramide, sphingomyelin, dihydrosphingomyelin, and sphingolipid ceramide *N*-deacylase (SCDase) were purchased from Avanti Polar Lipid Inc. (Alabaster, AL, USA). All other chemicals were commercially available.

### 4.2. Cell Culture

The C2C12 myoblast cell line was derived from primary cell culture. C2C12 myoblasts were cultured in Dulbecco’s modified Eagle’s medium (DMEM; Sigma-Aldrich) supplemented with 10% (*v*/*v*) fetal bovine serum (FBS; Gibco, Rockville, MD, USA) and 100 mg/mL penicillin-streptomycin. C2C12 myoblasts were growth in 100-mm cell culture dishes at 37 °C in a humidified 5% CO_2_ incubator. Cells were washed with PBS, and trypsin-EDTA was used to detach the cells from the cell culture plates.

### 4.3. Cell Viability

Cell viability was measured using an EZ-Cytox cell viability kit as previously described [57]. C2C12 myoblasts were seeded in 96-well cell culture plates (1 × 10^5^ cells/well) in DMEM supplemented with 10% (*v*/*v*) FBS at 37 °C for 24 h. The medium was then replaced with serum-free DMEM containing LA (0, 25, 50, 100, and 200 µM in 0.5% EtOH), and C2C12 myoblasts were incubated for 24 h. After supernatant suction and two washes, C2C12 myoblasts were treated with 1 mM H_2_O_2_ in serum-free DMEM for 2 h. The EZ-Cytox kit reagent was applied to all wells for 1 h, and the absorbance was measured at 450 nm with the FLx800 microplate reader.

### 4.4. Cytotoxicity

Cytotoxicity was measured using an EZ-Cytox cell viability kit as previously described [57]. C2C12 myoblasts were seeded in 96-well cell culture plates (1 × 10^5^ cells/well) in DMEM supplemented with 10% (v/v) FBS at 37 °C for 24 h. After removing the supernatant by suction, the C2C12 myoblasts were washed twice with PBS. The medium was replaced with serum-free DMEM containing LA (0, 25, 50, 100, and 200 µM with 0.5% EtOH), and C2C12 myoblasts were incubated for 24 h. All wells were treated with the EZ-Cytox kit reagent for 1 h, and absorbance was measured at 450 nm using the FLx800 microplate reader.

### 4.5. Evaluation of Apoptotic Cells

Apoptotic cells were analyzed as previously described [57]. C2C12 myoblasts were cultured on cover glass in 6-well cell culture plates with serum-free DMEM containing LA (0, 25, 50, 100, and 200 µM in 0.5% EtOH) for 24 h. After two washes with PBS, the medium was replaced with 1 mM H_2_O_2_ and incubated for 2 h. C2C12 myoblasts were fixed with 4% paraformaldehyde in PBS at room temperature for 30 min. Washed C2C12 myoblasts were mounted using mounting medium with DAPI. The cover glass containing the sample was then reversed and placed on a glass slide. Images for each sample were acquired using a Zeiss LSM710 confocal microscope. The acquisition parameters were kept constant for all images during confocal microscopy. Images were acquired at wavelengths of 358 and 461 nm. Apoptotic cells were counted using the ImageJ software. Typical apoptotic changes include condensation of chromatin, compaction along the periphery of the nucleus, and segmentation of the nucleus [57]. The rate of apoptosis was determined as the percentage of apoptotic nuclei per visual field. Apoptotic cells were calculated using the following equation: apoptosis (%) = [(number of apoptotic cells/number of total cells) × 100]. At least four fields of total and apoptotic cells were counted on each slide, for a total of 500 cells.

### 4.6. Quantitative Reverse-Transcriptase Polymerase Chain Reaction (qRT-PCR)

qRT-PCR was performed as previously described [26]. Total RNA was extracted according to the manufacturer’s instructions. The total RNA concentration was measured using a spectrophotometer (GE Healthcare, Buckinghamshire, UK). After measuring the RNA concentration, cDNA was synthesized according to the manufacturer’s instructions, and the extracted RNA was used as the template. qRT-PCR analysis was performed using SYBR Green 1 and a LightCycler 96 instrument (Roche, Basel, Switzerland). The following primers were used: mouse Atrogin-1/MAFbx sense, 5′-CCATCCTCTTTCTTGCCCGT-3′; mouse Atrogin-1/MAFbx antisense, 5′-ATCACTGTCCAACCTGGCTG-3′; mouse MuRF1 sense, 5′-TGGGACAGATGAGGAGGAGG-3′; mouse MuRF1 antisense, 5′-TTTACCCTCTGTGGTCACGC-3′; mouse GAPDH sense, 5ʹ-AGGTCGGTGTGAACGGATTTG-3ʹ; and mouse GAPDH antisense, 5′-TGTAGACCATGTAGTTGAGGTCA-3ʹ. The thermal cycling profile consisted of 40 cycles of denaturation (94 °C for 30 s), annealing (60 °C for 60 s), and extension (72 °C for 60 s). 

### 4.7. Detection of Intracellular ROS

Intracellular ROS were detected using the fluorescent dye DCF-DA, as previously described [57]. C2C12 myoblasts were seeded on a cover glass in 6-well cell culture plates and cultured in serum-free DMEM containing LA (0, 50, 100, and 200 µM with 0.5% EtOH) for 24 h. The medium was then replaced with 1 mM H_2_O_2_ for 2 h. After washing, the C2C12 myoblasts were incubated with 10 µM DCF-DA (dissolved in DMSO) in PBS at 37 °C for 30 min. 2′,7′-Dichlorofluorescin diacetate (DCF-DA) is a fluorogenic dye that measures hydroxyl, peroxyl, and other ROS activities within the cell. After diffusion into the cell, DCF- is deacetylated by cellular esterases to a non-fluorescent compound, which is later oxidized by ROS to 2′, 7′–dichlorofluorescein (DCF) [60,61,62]. The cells were then washed three times with PBS and fixed with 4% paraformaldehyde in PBS at room temperature for 30 min. C2C12 myoblasts were mounted using a mounting medium containing DAPI. Samples on the cover glass were reversed and placed on glass slides. Images were obtained using a Zeiss LSM710 confocal microscope (Carl Zeiss, Oberkochen, Germany). Images were acquired at a constant setting of the microscope. DAPI staining was detected at wavelengths of 358 nm and 461 nm, and DCF was identified at 485 nm and 535 nm. The mean GFP intensity was measured for cells greater than 10 μm in size and greater than 200 fluorescence units. The mean fluorescence intensity range was 5000–8000 units per cell, and data were expressed as the mean ± SD of H_2_O_2_ alone.

### 4.8. Immunoblot Analysis

C2C12 myoblasts were seeded in 6-well cell culture plates in DMEM with 10% (*v*/*v*) FBS at 37 °C for 24 h. At the end of the incubation, the supernatant was removed, and the cells were washed twice with PBS. Next, the medium was replaced with serum-free DMEM containing LA (0 and 200 µM in 0.5% EtOH), and C2C12 myoblasts were cultured for 24 h. The culture medium was replaced with 1 mM H_2_O_2_ and incubated for 2 h. GM/SM atrophy was induced by sciatic denervation. LA (2.5–5 mg/kg with 0.5% EtOH) was injected intramuscularly. C2C12 myoblasts were lysed, and proteins were extracted using the PRO-PREP protein extraction kit (iNtRON Biotechnology, Inc., Seongnam, Korea). Phosphatase inhibitors were used (1 mM Na_3_VO_4_, 10 mM NaF). For protein quantification, the Bradford assay kit (Bio-Rad, Hercules, CA, USA) was used according to the manufacturer’s instructions. Whole lysate proteins were analyzed by sodium dodecyl sulfate-polyacrylamide gel electrophoresis (SDS-PAGE) on 10–15% polyacrylamide gels. Proteins were transferred to polyvinylidene fluoride membranes (PVDF; Bio-Rad) using a Trans-Blot SD Semi-Dry Transfer Cell (Bio-Rad). The PVDF membrane was blocked for 1 h in Tris-buffered saline containing 0.1% Tween-20 (TBS-T) and 5% skim milk powder. The blocked membrane was incubated overnight at 4 °C with the appropriate primary antibody (1:2000). The primary antibodies used were anti-SOD1, anti-Bcl-2, anti-Bax, anti-HSP70, anti-p-FoxO1, anti-MuRF1, and anti-β-actin. After incubation with the primary antibody, the membranes were washed thrice with TBS-T and incubated for 2 h with each secondary antibody at room temperature (1:5000). Each protein signal was detected using enhanced chemiluminescence (ECL) reagent and the EZ-Western kit (DoGen, Seoul, Korea), and each image was analyzed using ImageQuant LAS4000 (GE Healthcare, Buckinghamshire, UK).

### 4.9. High-Performance Liquid Chromatography

Ceramides were quantified according to previously published procedures [57]. C2C12 myoblasts were lysed using 0.2 N NaOH and lipids were extracted using a methanol/chloroform/1 M NaCl solution (4:8:2.5, *v*/*v*/*v*). Dihydrosphingomyelin was used as an internal standard. The lipid layer was transferred to a new EP tube and dried. The dried samples were dissolved in 30 µL of methanol/chloroform (2:1 *v*/*v*). A solution of the dissolved lipid was spotted on an HPTLC silica gel plate for the separation of ceramide. To obtain sphingosine from ceramide, SCDase was added to the reaction buffer (containing 25 mM Tris-HCl buffer, 1% sodium chlorate, and 15% fatty acid-free bovine serum albumin, pH 7.5). The converted sphingosine was analyzed using HPLC following *ortho*-phthaldialdehyde derivatization.

### 4.10. Animal Experiment

The animal experiments were performed as previously described [57]. Four-week-old male C57BL/6 mice were purchased from Orient Bio (Gangneung, South Korea). The animal experimental protocol was approved by the Institutional Animal Care and Use Committee (IACUC, YWC-160217-1) of Yonsei University (Wonju, Korea). The mice were kept in wired cages at an ambient temperature of 20–22 °C and a humidity of 40–50%. An attempt was made to minimize pain in the animals. The sciatic nerve in the right leg of each mouse was surgically removed to induce immobilization. These mice exhibited muscle atrophy induced by sciatic denervation for seven days. Then, LA (2.5–5 mg/kg with 0.5% EtOH) was injected intraperitoneally 10 times during a 2-week period. The mice were sacrificed 21 days after sciatic denervation. 

### 4.11. Micro-Computed Tomography (micro-CT)

Micro-CT was performed as previously described [57]. Images for the muscle in the tibia of each mouse (*n* = 5) were acquired on day 21 after induced muscle atrophy using micro-CT (SkyScan 1076, Bruker AXS, Karlsruhe, Germany). The scan parameters were set to 18 µm resolution, 100 kV voltage, 100 µA current, 2065 ms exposure time, 1 mm thickness of the aluminum filter, and 0.7° rotation step. During rotation and scanning, all mice were anesthetized. Beam-hardening errors were corrected to improve the quality of the micro-CT images using flat-field correction before scanning and beam-hardening correction during reconstruction. For the evaluation of muscle volume, two-dimensional (2D) and three-dimensional (3D) models of muscle were realized using CT-Analyzer 1.11 (CT-An 1.11; Bruker). 

### 4.12. Statistical Analysis

The experimental results are expressed as the mean ± standard deviation. Statistical analysis of all data was performed using one-way analysis of variance (one-way ANOVA) and multiple comparisons followed by a Tukey–Kramer post-hoc analysis; *p* < 0.05, *p* < 0.01, and *p* < 0.001 were considered statistically significant, highly significant, and very highly significant, respectively.

## 5. Conclusions

In this study, LA suppressed apoptosis induced by ROS in vitro. Moreover, muscle atrophy at the molecular level due to sciatic denervation coincided with the results of the in vitro experiment, implicating oxidative stress in muscle atrophy. LA has been shown to significantly prevent and recover this condition. Therefore, LA is a promising candidate compound for treating patients with muscle atrophy. We believe that this study will contribute to future studies investigating muscle atrophy.

## Data Availability

The data used to support the findings of this study are available from the corresponding author upon request.
